# Sequencing and *de novo* assembly of the Asian gypsy moth transcriptome using the Illumina platform

**DOI:** 10.1590/1678-4685-GMB-2015-0257

**Published:** 2016-10-20

**Authors:** Fan Xiaojun, Yang Chun, Liu Jianhong, Zhang Chang, Li Yao

**Affiliations:** Department of Biological and Pharmaceutical Engineering, College of Chemistry and Chemical Engineering, Taiyuan University of Technology, Taiyuan, Shanxi, China

**Keywords:** Asian gypsy moth, chitin metabolism, transcriptome, unigenes

## Abstract

The Asian gypsy moth (*Lymantria dispar*) is a serious pest of forest and shade trees in many Asian and some European countries. However, there have been few studies of *L. dispar* genetic information and comprehensive genetic analyses of this species are needed in order to understand its genetic and metabolic sensitivities, such as the molting mechanism during larval development. In this study, high-throughput sequencing technology was used to sequence the transcriptome of the Asian subspecies of the gyspy moth, after which a comprehensive analysis of chitin metabolism was undertaken. We generated 37,750,380 high-quality reads and assembled them into contigs. A total of 37,098 unigenes were identified, of which 15,901 were annotated in the NCBI non-redundant protein database and 9,613 were annotated in the Swiss-Prot database. We mapped 4,329 unigenes onto 317 pathways using the Kyoto Encyclopedia of Genes and Genomes Pathway database. Chitin metabolism unigenes were found in the transcriptome and the data indicated that a variety of enzymes was involved in chitin catabolic and biosynthetic pathways.

## Introduction

The gypsy moth, *Lymantria dispar* (Linnaeus) (Lepidoptera: Lymantriidae), is one of the most destructive polyphagous pests in forests, agro-ecosystems, shade trees and shrubs, with more than 500 host plants identified so far (Matsuki *et al*., 2010; Peric-Mataruga *et al*., 2014). Subspecies of the gypsy moth occur in Europe, North Africa, Asia and North America (Manderino *et al*., 2014). The ‘Asian form’ is characterized by females that fly actively, whereas only non-flying females are known in Europe and North America. The ‘Asian form’ also has a wider host range than those in Europe and North America (Roy *et al*., 1995). Asian gypsy moth has defoliated an average of two million acres of forest per year over the past 20 years in northern and eastern China, causing a significant economic impact (Sun *et al*., 2014). A substantial effort has been made to slow the spread of the moth, including attempts to fully control this insect pest. However, the range of this species has been continued to expand into central China. Control measures that have been implemented in China to reduce the spread of this plant pathogen include the release of natural enemies such as *Coccygomimus disparis*, the application of chemical pesticides (mainly organophosphates, pyrethriods and carbamates) and chemical pheromones to disrupt mating (Disparlure), biological control (using *Bacillus thuringiensis*), and the use of transgenic host plants. Pesticides remain the most rapid and effective strategy for combating the pest, but gypsy moth insecticide resistance is a growing concern although the underlying causes are currently unknown. Limited genetic information exists for gypsy moths in general, especially the Asian subspecies. Comprehensive analyses are therefore needed to reveal genetic sensitivities of the pest, improve bioinsecticide selection, recognize genes that are determinants of disease and resistance development, and facilitate targeted pest management (Sparks *et al*., 2013).

Chitin is a polysaccharide long-chain polymer comprised of β-(1,4)-N-acetylglucosamine residues and is a vital component of the insect cuticle and peritrophic membrane (PM) (Kramer *et al*., 1995). During insect growth and development, the cuticle and PM must be degraded periodically and then be replaced in order to allow for growth, maturation and repair (Zakariassen *et al*., 2010; Zhang *et al*., 2011). One important characteristic of the epidermis is that two closely related processes (the degradation and synthesis of chitin) occur almost simultaneously during metamorphosis (Gu *et al*., 2013). Chitin is digested to GlcNAc in the larval cuticle by a binary enzyme system composed of endochitinase and exochitinase. The synthesis of chitin relies on two key enzymes, chitin synthase (CHS) and glutamine-fructose-6-phosphate aminotransferase (GFAT), which provide the GlcNAc precursor for the chitin biosynthetic pathway (Kramer and Koga, 1986; Kramer *et al*., 1995). Although RNAseq-based transcriptome analyses of *Spodoptera litura* (Lepidoptera) and the *Lymantria dispar* (*L. Dispar*) larval midgut in response to infection by *B. thuringiensis* have been undertaken (Gu *et al*., 2013), more comprehensive descriptions of the transcriptomes of other (uninfected) lepidopteran species would be beneficial in elucidating chitin-related metabolic processes in these organisms, thereby helping scientists to find potentially new targets for biocontrol.

Since the sequencing of large genomes can be expensive, transcriptome analysis provides a useful, cost-effective method of discovering new genes and provides information on gene expression, gene regulation and amino acid content of proteins (Huse *et al*., 2007; Novaes *et al*., 2008). In this study, we used transcriptome sequencing and *de novo* gene assembly to examine chitin metabolism in Asian *L. dispar*.

## Materials and Methods

### Insect rearing and isolation of total RNA

Asian *L. dispar* eggs were obtained from the Forest Ecology and Conservation of the Environment, Chinese Academy of Forestry (Beijing, China). The eggs were placed in an incubator for incubation and rearing. Whole insect samples (gut and epidermis) were immediately frozen and stored in liquid nitrogen until analysis. Total RNA was extracted from these samples using RNAiso reagents (Takara Biotechnology Co. Ltd., Dalian, China) (Fan *et al*., 2015). The quality and quantity of total RNA were determined using gel electrophoresis (Beijingliuyi, Beijing, China) and a Multiskan GO microplate spectrophotometer (Thermo Scientific, Waltham, MA, USA). Equal quantities of high-quality RNA from each sample were pooled for cDNA synthesis.

### mRNA-seq library construction for Illumina sequencing

The mRNA-seq library was constructed using an mRNA-Seq sample preparation kit (Cat. no. RS-930-1001, Illumina Inc., San Diego, CA, USA) according to the manufacturer's instructions. Poly-(A) mRNA was isolated from total RNA using poly-T oligo-attached magnetic beads. The mRNA was fragmented with an RNA fragmentation kit (Ambion, Austin, TX, USA) before cDNA synthesis in order to avoid priming bias. The cleaved RNA fragments were transcribed into first-strand cDNA using reverse transcriptase (Invitrogen, Carlsbad, CA, USA) (Invitrogen) and random hexamer primers, followed by second-strand cDNA synthesis using DNA polymerase I and RNase H (Invitrogen). The double-stranded cDNA was end-repaired using T4 DNA polymerase (New England Biochemicals - NEB, Ipswich, MA, USA), Klenow fragment (NEB) and T4 polynucleotide kinase (NEB). Base addition was done using Klenow 39 to 59 exopolymerase (NEB) to prepare the DNA fragments for ligation to the adaptors. The products of ligation were purified using a MinElute PCR purification kit (QIAGEN, Düsseldorf, Germany) according to the manufacturer's instructions, and eluted in 10 mL of QIAGEN EB buffer. The eluted adaptor-ligated fragments of the ligation reaction were separated on an agarose gel to select a size range of templates for downstream enrichment. The desired range of cDNA fragments was excised and retrieved using a gel extraction kit (Axygen Biosciences, San Francisco, CA, USA). PCR was used for selective enrichment and amplification of the cDNA fragments using a Phusion master mix (NEB) with two primers (primers PE 1.0 and PE 2.0) generated with an mRNASeq sample preparation kit (Illumina). The amplified products were purified using a QIAquick PCR purification kit (QIAGEN) according to the manufacturer's instructions and eluted in 30 mL of QIAGEN EB buffer. Libraries were prepared from a 150-200 bp size pool after adaptor ligation and agarose gel separation. After accurate quantitation (Qubit) of the 150-200 bp size pool, bridge PCR was done on the surface of the flow cell to amplify DNA fragments as a single DNA molecule cluster (this process was done with a Cluster Station). Each attached DNA fragment underwent multiple rounds of amplification to create a cluster of identical DNA fragments. With each sequencing cycle, a fluorescently-labeled base was incorporated into each fragment in the cluster and images of the flow cell surface were captured (Bentley *et al*., 2008). Image analysis algorithms are applied during the first few cycles to identify the positions of individual clusters that were then monitored through subsequent cycles to generate sequence data. The ability to successfully read sequences from a lane is critically dependent on the ability to correctly map the cluster coordinates (Krueger *et al*., 2011).

### Sequence data analysis and assembly

Pair-end raw reads were trimmed with BWA trimming mode at a threshold of Q20 (P = 0.01) as implemented by SolexaQA (Cox *et al*., 2010). The qualified reads were extended into contigs with Trinity software through the overlap between the sequences and the contigs then connected into transcript sequences based on the paired-end information of the sequences, which recovers full-length transcripts across a broad range of expression levels with a sensitivity similar to methods that rely on genome alignments (Grabherr *et al*., 2011). The overlap settings used for this assembly were 24 bp and 80% similarity, and the group pairs distance was set to 500 (maximum length expected between fragment pairs); all the other parameters were set to their default values. The longest transcription from the potential assembled component alternative splicing transcripts was selected as the unigene sequences of our sample. We quantified the transcript levels in reads per kilobase of exon per million mapped reads (RPKM) (Mortazavi *et al*., 2008). The RPKM measure of read density reflects the molar concentration of a transcript in the starting sample by normalizing for RNA length and for the total number of reads in the measurement.

### Sequence annotation

The predicted amino acid sequences encoded by the unigene sequences were annotated using the following protein databases: National Center for Biotechnology Information (NCBI) non-redundant protein (Nr) database and Swiss-Prot database using BLASTx (BLAST, the basic local alignment search tool) with an E-value cutoff of < 10^−5^. Gene names were assigned to each assembled sequence based on the best BLAST hit (highest score). Searches were limited to the first 10 significant hits for each query in order to increase computational speed. Open reading frames (ORFs) were predicted using the "getorf" program of the EMBOSS software package (Rice *et al*., 2000), with the longest ORF extracted for each unigene.

To annotate the assembled sequences with Gene Ontology (GO) terms describing biological processes, molecular functions and cellular components, the Swiss-Prot BLAST results were imported into Blast2GO (Conesa *et al*., 2005; Conesa and Götz, 2008), a software package that retrieves GO terms and allows gene functions to be determined and compared. These GO terms were assigned to query sequences and produced a broad overview of groups of genes catalogued in the transcriptome for each of the three ontology vocabularies (biological processes, molecular functions and cellular components). The annotation obtained was enriched and refined using ANNEX (Myhre *et al*., 2006; Ye *et al*., 2006).

The unigene sequences were also aligned to the Clusters of Orthologous Group (COG) database to predict and classify functions (Tatusov *et al*., 2000). Kyoto Encyclopedia of Genes and Genomes (KEGG) pathways were assigned to the assembled sequences using the online KEGG Automatic Annotation Server (http://www.genome.jp/kegg/kaas/). The bi-directional best hit method was used to obtain a KEGG orthology (KO) assignment. The output of the KEGG analysis included KO assignments and KEGG pathways that were populated with the KO assignments.

## Results

### Asian *Lymantria dispar* transcriptome sequencing and *de novo* assembly

**Figure 1:**
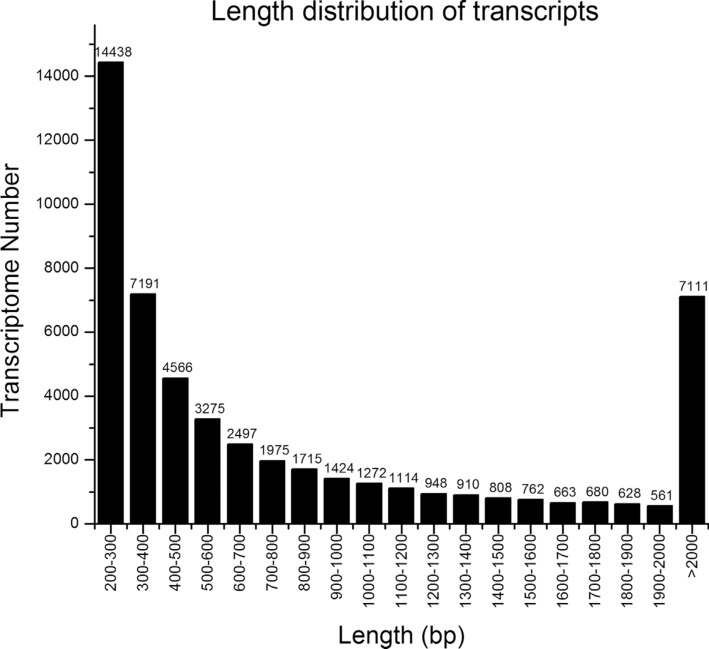
Length distribution of *Lymantria dispar* transcripts.

**Figure 2:**
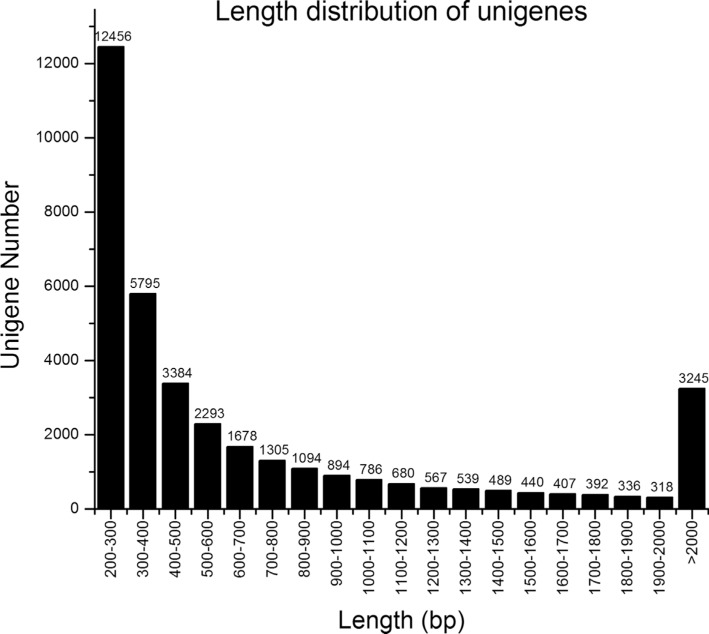
Length distribution of *Lymantria dispar* unigenes.

Sequence analysis and assembly were used to obtain a global overview of the transcriptome and gene activities of Asian gypsy moth at the nucleotide level. A mixed cDNA sample representing gut and epidermis of *L. dispar* was prepared and sequenced using an Illumina genome analyzer. RNA-Seq transcriptomic profiling of the larval stage of the Asian gypsy moth generated approximately 71.8 million clean short reads with a mean size of 95.67 bp that were assembled into contigs. After paired-end joining and gapfilling, the contigs were further assembled into 37,098 unigenes with a mean size of 787.96 bp (Table 1). The high quality reads produced in this study were deposited in the NCBI SRA database (accession number: SRX1520900). Using the Trinity *de novo* assembly program, next-generation short-read sequences were assembled into 52,538 transcripts, with an N50 length of 1,891 bp and a mean length of 1006.79 bp (Table 2). The length distribution of transcripts is shown in Figure 1. The transcripts were subjected to cluster and assembly analysis. A total of 37,098 unigenes were obtained, of which 8,208 genes (22.13%) were > 1 kb in size. The length distribution of the unigenes is shown in Figure 2; more than 15,488 unigenes (41.75%) were > 500 bp in size.

**Table 1:** Output statistics for the *L. dispar* transcriptome sequencing process.

Total number of raw reads	75,500,760
Average raw read length (bp)	100
Total number of clean reads	71,801,515
Average clean read length (bp)	95.67
Q20 bases ratio (%)	89.37

**Table 2:** Statistics of assembled sequences.

	All numbers	All length (bp)	Mean length (bp)	N50 (bp)
Transcript	52538	52,894,995	1006.79	1891
Unigene	37098	29,231,915	787.96	1391

### Functional annotation

Several complementary approaches were used to annotate the assembled sequences. The unigenes were annotated by aligning them with those deposited in diverse protein databases, including the Nr database, UniProt/Swiss-Prot, KEGG, COG of proteins and UniProt/TrEMBL. The best alignment was selected from the matches with an E-value > 10^−5^. The overall functional annotation is depicted in Table 3. Initially, a sequence similarity search was run against the NCBI Nr and Swiss-Prot protein databases using the BLAST algorithm and specifying E-values < 10^−5^. There were 15,901 unigenes that matched in the Nr database and 9,613 were similar to proteins in the Swiss-Prot database; 15,826 unigene matches were found in the TrEMBL database.

**Table 3:** Functional annotation of the *L. dispar* transcriptome.

Unigene no. (%)	NR	SWISS-PROT	TREMBL	PFAM	KOG	KEGG
37098	15901	9613	15826	5750	7303	4329
(100)	(42.86)	(25.91)	(42.66)	(15.50)	(19.69)	(11.67)

### GO classification

GO classification based on sequence homology revealed that 12,290 of the assembled unigenes were classified into 62 functional groups. Three major categories (biological process, cellular component and molecular function) were assigned to 47,166, 35,597 and 16,594 GO terms, respectively. In the "biological process" category, the unigenes related to "metabolic processes" (18.75%), "cellular processes" (21.42%), "single-organism process" (13.97%), "biological regulation" (9.90%), "regulation of biological process" (9.17%), "response to stimulus" (7.61%) and "multicellular organismal process" (6.48%) were predominant. The most abundant classes were "cell parts" (20.67%) and "cell" (20.67%), "organelle" (15.26%), "membrane" (9.63%), and "organelle part" (8.34%). Most of the genes in the "molecular function" category were involved in "binding" (20.24%) and "catalytic activities" (15.78%) (Table 4).

**Table 4:** Functional annotation of assembled sequences based on gene ontology (GO) categorization.

Gene Ontology	Categories	% unigenes
Biological process	Metabolic processes	18.75
	Cellular processes	21.42
	Single-organism process	13.97
	Biological regulation	9.90
	Regulation of biological process	9.17
	Response to stimulus	7.61
	Multicellular organismal process	6.48
Cellular component	Cell parts	20.67
	Cell	20.67
	Organelle	15.26
	Membrane	9.63
	Organelle part	8.34
Molecular function	Binding	20.24
	Catalytic activities	15.78

### KOG classification

**Figure 3:**
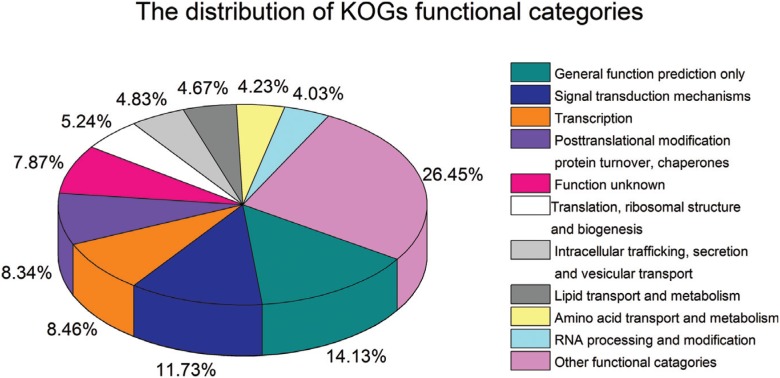
Clusters of orthologous group (KOG) classification.

KOG classification showed that 7,302 unigenes were clustered into 25 functional categories. "General function prediction only" (14.13%) was the major KOG category, followed by "signal transduction mechanisms" (11.73%), "transcription" (8.46%), "posttranslational modification, protein turnover, chaperones" (8.34%), "translation, ribosomal structure and biogenesis" (5.24%), "intracellular trafficking, secretion and vesicular transport" (4.83%) and "lipid transport and metabolism"(4.67%) (Figure 3).

### KEGG classification

**Figure 4:**
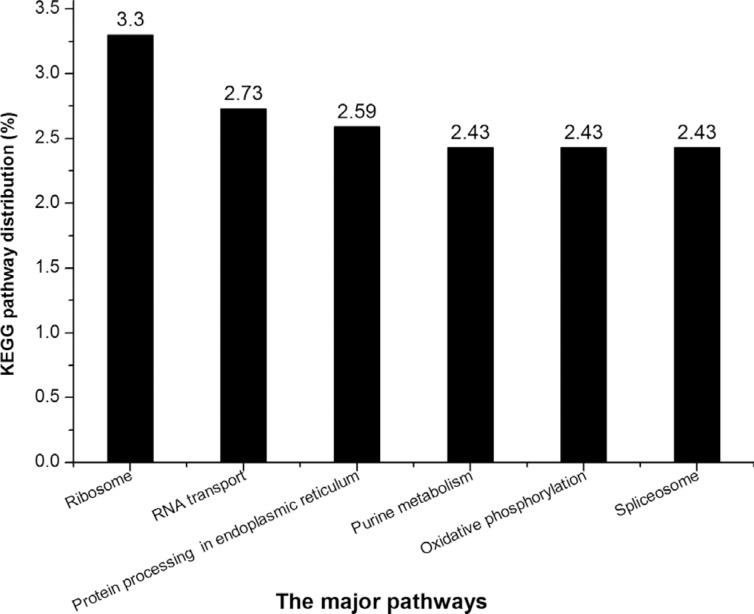
KEGG biochemical maps for *Lymantria dispar*.

KEGG analysis showed that 4,329 unigenes were assigned to 317 pathways. The major pathways that contained > 100 unigenes were ribosome (ko03010) (143 unigenes, accounting for 3.30%), RNA transport (ko03013) (118, 2.73%), protein processing in endoplasmic reticulum (ko04141) (112, 2.59%), purine metabolism (ko00230) (105, 2.43%), oxidative phosphorylation (ko00190) (105, 2.43%) and spliceosome (ko03040) (105, 2.43%) (Figure 4).

### NCBI non-redundant classification

**Figure 5:**
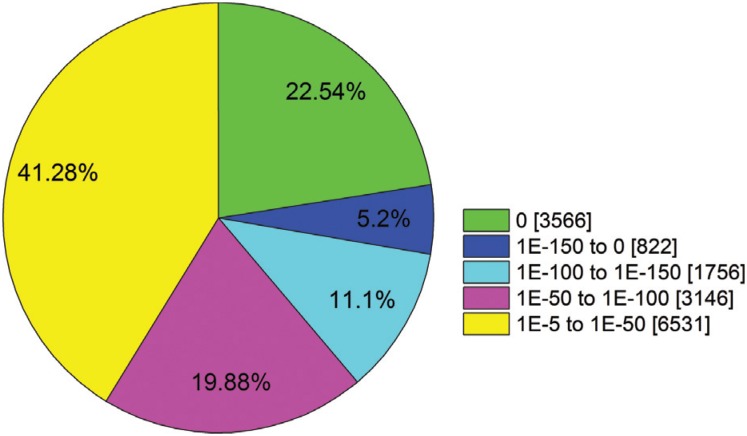
E-value distribution of the top Blastx hits against the non-redundant (Nr) protein database for each unigene.

Of the aligned unigenes, 58.72% showed very strong homology to sequences in the non-redundant (Nr) protein database (E-value < 1.0E^−50^), while the remaining 41.28% showed weak homology (E-value between 1.0E^−5^ and 1.0E^−50^) (Figure 5).

**Figure 6:**
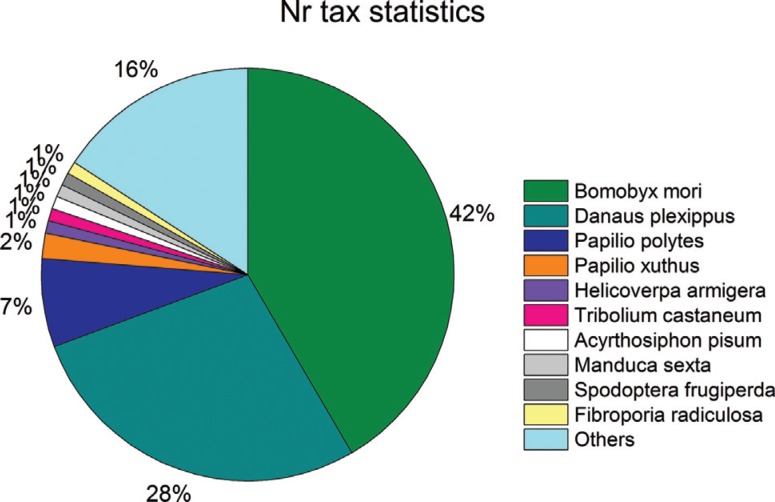
Similarity analysis based on the BLAST best hit.

The similarity distribution of aligned unigenes compared to sequences in the Nr database showed that 71.3% of unigenes had a similarity > 60%, 24.08% of the unigenes had a similarity between 40% and 60%, and only 4.62% of unigenes had < 40% similarity with Nr sequences (Table 5). In the species distribution of similar sequences in the Nr database, ~84% of unigenes matched sequences from just ten insect species, namely *Bombyx mori* (42% of unigenes), *Danaus plexippus* (28%), *Papilio polytes* (7%), *Papilio xuthus* (2%), *Helicoverpa armigera* (1%), *Tribolium castaneum* (1%), *Acyrthosiphon pisum* (1%), *Manduca sexta* (1%), *Spodoptera frugiperda* (1%) and *Fibroporia radiculosa* (1%) (Figure 6). In summary, homologous sequence annotation information could be obtained for the majority of unigenes. These results also demonstrated that the accuracy of the assembled transcripts was very high and that our sequencing strategy was efficient.

**Table 5:** Similarity distribution of *L. dispar* unigenes with sequences in the Nr database.

	Unigenes	
Similarity range (%)	Number (Nr)	Percent (%)
20–40	731	4.62
40–60	3809	24.08
60–80	6058	38.29
80–100	4985	31.51
100	238	1.50

## Discussion

The mean unigene length identified in the Asian gypsy moth (788 bp) was much longer than that of *Leptinotarsa decemlineata* (538 bp), and the N50 length of the *L. dispar* transcriptome (1391 bp) was significantly longer than that of *L. decemlineata* (570 bp) (Kumar *et al*., 2014). As N50 length is commonly used to evaluate the accuracy of an assembly (Lander *et al*., 2001), our results suggest that the quality of our assembly was high. Although a previous study had already reported a transcriptomic analysis for gypsy moth (Cao *et al*., 2015), there are considerable differences between the two databases. In the present study, the number of unigenes was 37,098, with a mean length of 787.96 bp and an N50 length of 1391 bp, compared to corresponding values of 62,063 unigenes, 669 bp and 993 bp in the report by Cao *et al*. (2015). The additional data provided by our database will enrich the current transcriptomic information for the Asian gypsy moth.

Based on the GO classification, *L. dispar* unigenes were mainly related to cellular processes (21.42%), metabolic processes (18.75%), single-organism processes (13.97%) and biological regulation (9.90%). The high number of unigenes related to "cellular process" and "metabolic processes" indicated that the Asian gypsy moth is metabolically active. Based on sequence homology, the results revealed that 24 unigenes were related to the chitin catabolic process (GO: 0006032) and 86 unigenes were related to the chitin metabolic process (GO: 0006030); only three unigenes related to the chitin biosynthetic process (GO: 0006031) were identified. These results suggest that the chitin metabolic process is a sophisticated and dynamically cyclic process containing unique genetic information on chitin metabolism in this moth. One important feature of the epidermis was that two closely-related processes, namely, chitin synthesis and degradation, occurred almost simultaneously during metamorphosis. Chitin is digested to GlcNAc in the larval cuticle by a binary enzyme system composed of endochitinase (commonly referred to as chitinase) and exochitinase (β-N-acetylglucosaminidase). The former enzyme hydrolyzes chitin into oligosaccharides while the latter further degrades the oligomers to monomers. The synthesis of chitin relies on two key enzymes, chitin synthase (CHS) and glutamine-fructose-6-phosphate aminotransferase (GFAT), which provides the GlcNAc precursor for the chitin biosynthetic pathway. Insect epidermal metamorphosis is a complicated process involving multiple genes and pathways (Gu *et al*., 2013). In our study, the number of unigenes related to chitin degradation was much higher than for chitin biosynthesis and involved a relatively high RPKM value. For example, comp23115_c0_seq1 in the chitinase pathway was expressed at the highest level (RPKM = 152.20). Overall, these results were consistent with Liang's study in 2010, which suggested that the synthesis of new chitin was concomitant with the degradation of old chitin, with degradation products possibly being reused in the synthesis of new chitin (Liang *et al*., 2010). Additional studies involving intensive molecular and proteomic analyses are required to validate these gene functions.

## Conclusion

This is the first comprehensive transcriptome analysis of the Asian gypsy moth from mixed tissues (gut and epidermis) using the Illumina platform. Our analysis provided 37,098 unigenes, of which 42.86% were aligned to sequences in the Nr database. Although a reference genome sequence for *L. dispar* remains unavailable, we identified a large number of candidate genes potentially involved in growth, development, pupation and molting that are worthy of further investigation. These data will be useful for future explorations of the mechanism of molting in *L. dispar*.
